# Supplementary material for: A Dynamical Systems Approach to Characterizing Brain–Body Interactions during Movement: Challenges, Interpretations, and Recommendations
Source: Sensors (Basel). 2023 Jul 11;23(14):6296. doi: 10.3390/s23146296 (PMC10385586; doi:10.3390/s23146296)
Supplement: Supplementary file 1 [file sensors-23-06296-s001.zip › sensors-2407885-supplementary.pdf]

SUPPLEMENTARY MATERIALS

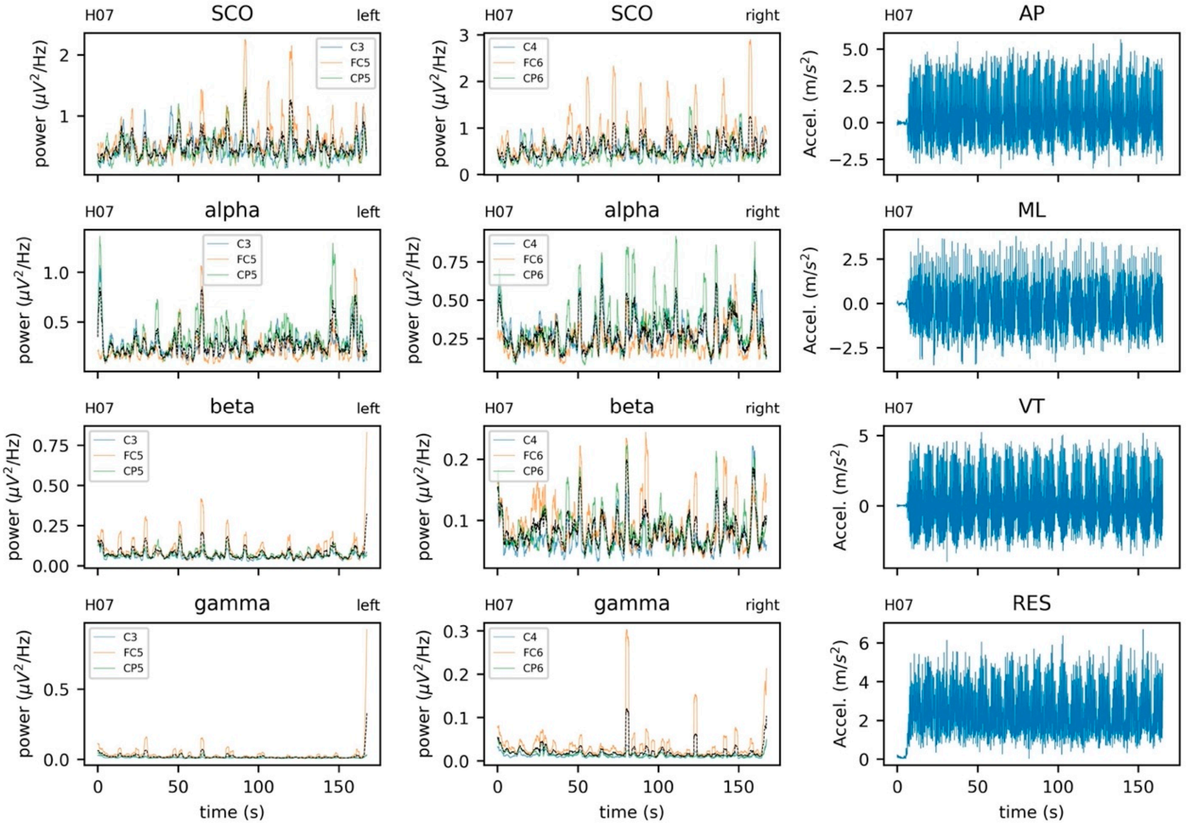

Figure S1. Electroencephalogram (EEG) and center of mass (COM) data from a single subject. EEG data includes alpha, beta, gamma, and combined theta/delta (SCO) bands. COM data includes anteroposterior (AP), mediolateral (ML), vertical (VT), and resultant (RES) accelerations.

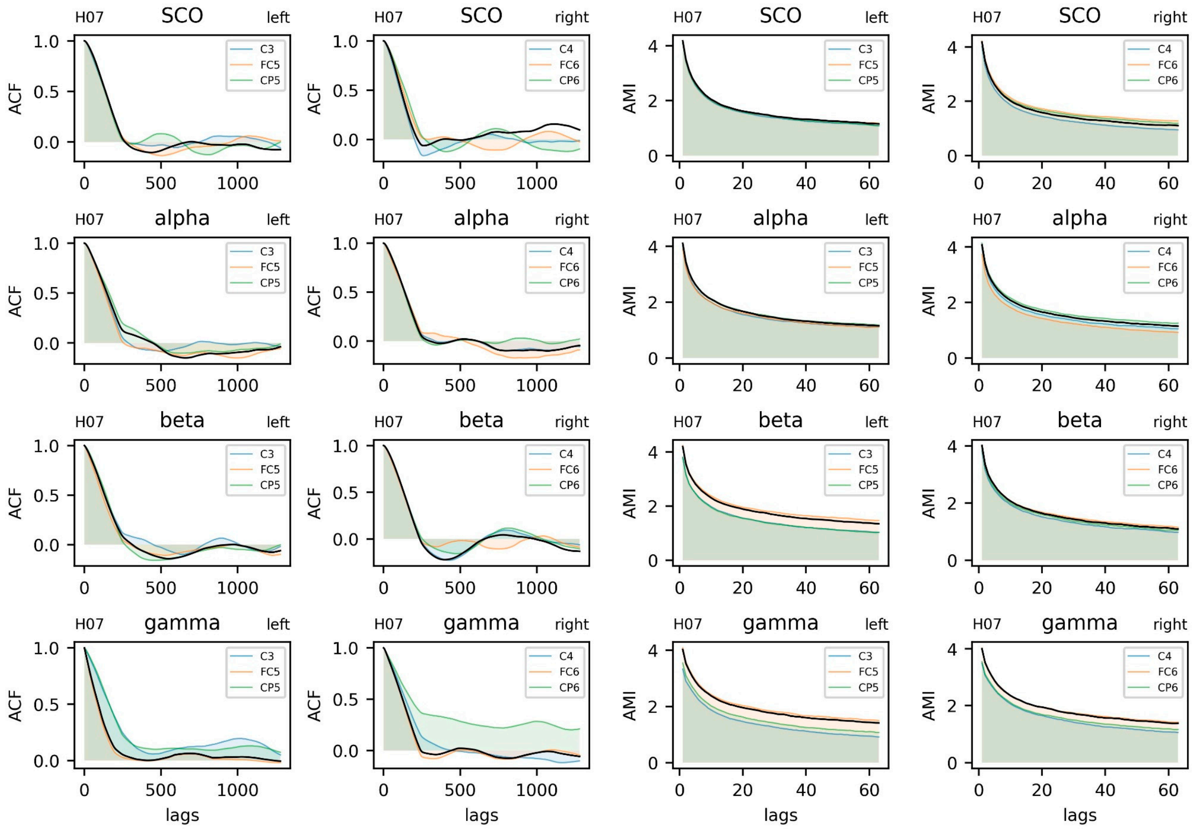

Figure S2. Autocorrelation and average mutual information functions for electroencephalogram (EEG) and center of mass (COM) data from a single subject. EEG data includes alpha, beta, gamma, and combined theta/delta (SCO) bands. COM data includes anteroposterior (AP), mediolateral (ML), vertical (VT), and resultant (RES) accelerations.

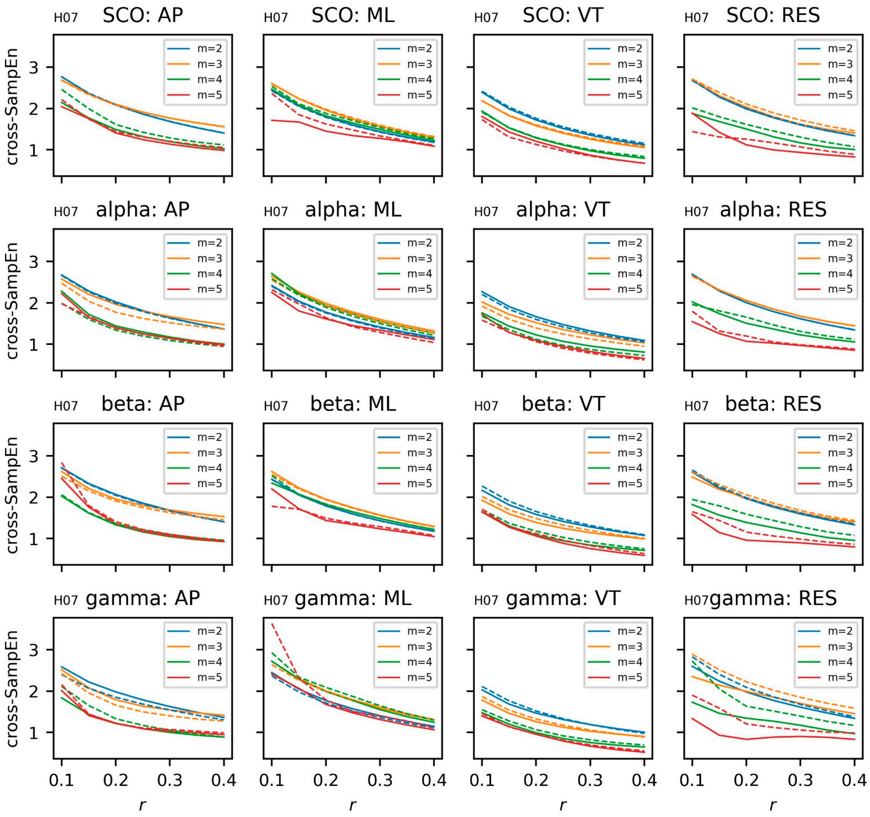

Figure S3. Cross-sample entropy for electroencephalogram (EEG) data from a single subject. EEG data includes alpha, beta, gamma, and combined theta/delta (SCO) bands. COM data includes anteroposterior (AP), mediolateral (ML), vertical (VT), and resultant (RES) accelerations. [m=(2, 3, … 5), r=(0.1, 0., … 0.40), τ=16]

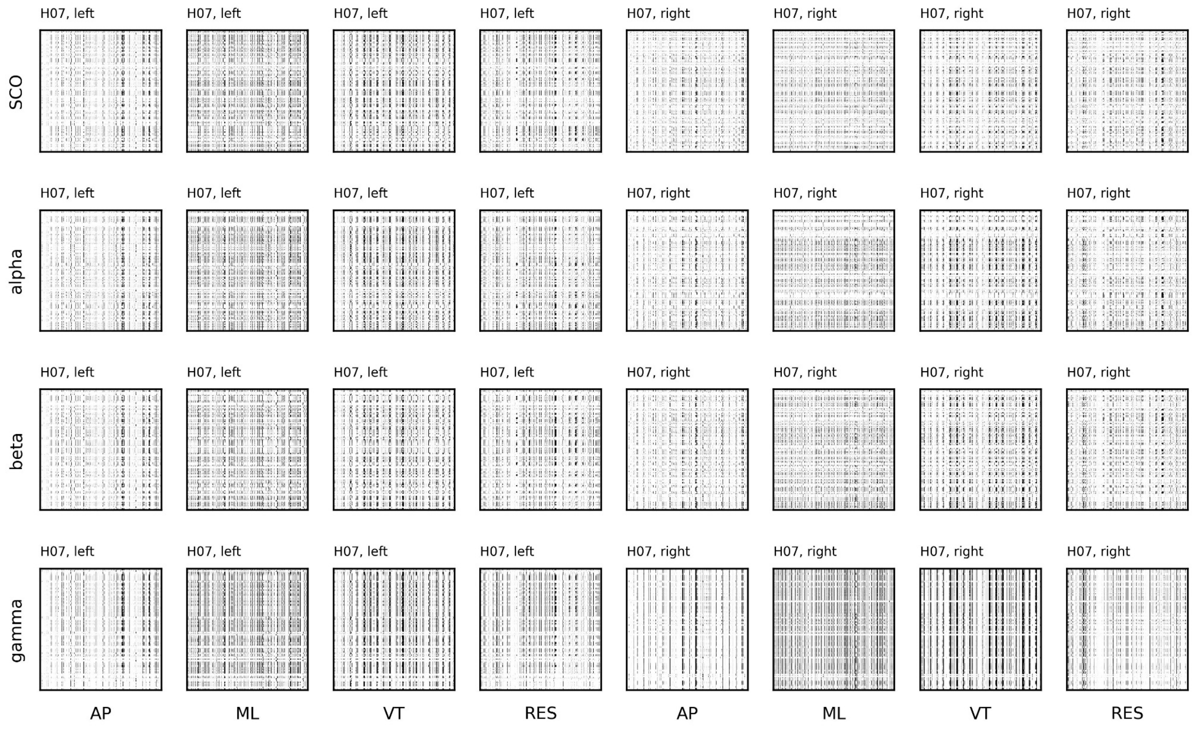

Figure S4. Cross-recurrence quantification (cRQA) for electroencephalogram (EEG) and center of mass (COM) data from a single subject. EEG data includes alpha, beta, gamma, and combined theta/delta (SCO) bands. COM data includes anteroposterior (AP), mediolateral (ML), vertical (VT), and resultant (RES) accelerations. [m=3, r=0.2, τ=16, tw=1]
